# A Low-Cost Vision–GPS Framework for the Unified Mapping of Vertical and Horizontal Road Assets Using Deep Learning

**DOI:** 10.3390/s26134042

**Published:** 2026-06-25

**Authors:** Domenico Profumo, Raza Akbar, Laura Fiorella, Luca Fredianelli, Elena Ascari, Francesco D’Alessandro, Francesco Fidecaro, Gaetano Licitra

**Affiliations:** 1 iPOOL s.r.l., Via Antonio Cocchi 3, 56121 Pisa, Italy; domenico.profumo@i-pool.it (D.P.); francesco.dalessandro@i-pool.it (F.D.); 2 Department of Physics “E. Fermi”, University of Pisa, Largo Bruno Pontecorvo 3, 56127 Pisa, Italy; raza.akbar@df.unipi.it (R.A.); francesco.fidecaro@unipi.it (F.F.); 3 Institute for Chemical-Physical Processes, Italian Research Council (CNR-IPCF), Via Moruzzi 1, 56100 Pisa, Italy; laura@swot.it (L.F.); elena.ascari@cnr.it (E.A.); 4 Pisa Department, Environmental Protection Agency of Tuscany (ARPAT), Via Vittorio Veneto 27, 56127 Pisa, Italy; g.licitra@arpat.toscana.it

**Keywords:** road asset mapping, traffic sign detection, instance segmentation, deep learning, YOLO, Intelligent Transportation Systems (ITS)

## Abstract

Automated mapping of vertical traffic signs and horizontal road markings is essential for road safety and Intelligent Transportation Systems (ITS). Traditional methods are labor-intensive, while existing automated solutions often lack a unified approach or are proprietary, limiting research accessibility and reproducibility. This paper presents a comprehensive framework for identifying these assets using a low-cost, vehicle-mounted action camera. A distance-aware frame extraction strategy is introduced to minimize data redundancy and ensure high spatial diversity. Specific strategies address the class imbalance inherent in real-world driving, ensuring robust detection for infrequent sign categories. Deep learning models handle the distinct geometries of vertical and horizontal assets, employing segmentation-based annotation for irregular road markings. Experimental results show high performance, with leading YOLO-based architectures achieving an F1-score of 0.92 for vertical signage and 0.96 for horizontal markings. By transforming raw visual data into structured georeferenced information, this framework facilitates the generation of High-Definition (HD) maps and digital inventories, supporting road authorities in proactive maintenance planning and regional road safety assessments.

## 1. Introduction

Road infrastructure is a fundamental component of safe and efficient transportation systems, where traffic control devices such as vertical signs and horizontal road markings serve as safety-critical elements. According to the World Health Organization [1], road traffic crashes cause approximately 1.19 million deaths annually worldwide, with tens of millions of additional non-fatal injuries, confirming that road safety remains a major global challenge. In this context, traffic signs and pavement markings provide regulatory, warning, and guidance information that supports both driver decision-making and vehicle positioning. When these elements are faded, occluded, or poorly maintained, their ability to support safe driving is significantly reduced. This safety relevance is reflected in international transportation practice. Continuous monitoring and mapping of these assets are therefore important not only for conventional maintenance, but also for digital road inventories, intelligent transportation systems, and HD map generation for advanced mobility applications. HD maps are widely recognized as a key enabling component for autonomous and connected mobility, since they provide high-resolution spatial information required for localization, perception support, and infrastructure-aware navigation [2]. Automated road-asset mapping is already an active technological and commercial field. For instance, Mobileye’s REM framework [3] builds semantic road maps from production vehicles, while Cyclomedia [4] provides commercial services for traffic sign and pavement striping inventory and condition assessment. However, these solutions are generally proprietary, often rely on large-scale mapping ecosystems or specialized sensing infrastructures, and are not necessarily easy to reproduce or transfer to low-cost research and road-management contexts. From a scientific perspective, road-asset detection has been investigated through both high-end mobile mapping systems and vision-based image analysis. Modern mobile mapping systems typically integrate GNSS/IMU units, calibrated cameras, and LiDAR [5]. Although these systems provide accurate and information-rich data, they are expensive and involve substantial acquisition, storage, and processing requirements. Consequently, low-cost image-based approaches have attracted increasing attention in scenarios requiring frequent, scalable, and easily deployable road surveys [6]. Recent low-cost edge-AI systems have also demonstrated the feasibility of using compact vision-based sensing platforms for real-time road-traffic monitoring tasks, further supporting the adoption of affordable sensing architectures for transport-infrastructure applications [7]. Within computer vision, early benchmark efforts such as the German Traffic Sign Recognition Benchmark [8] and the German Traffic Sign Detection Benchmark [9] contributed to the standardization of traffic-sign evaluation. Later, more diverse datasets such as TT100K [10] and the Mapillary Traffic Sign Dataset [11] extended the problem to a wider variety of classes and geographical contexts. In parallel, the rapid development of YOLO-based object detection architectures has strongly influenced traffic sign detection and recognition research, as highlighted by recent systematic reviews that document the widespread adoption of YOLO models for real-time traffic-sign perception tasks [12]. Despite this progress, several persistent limitations remain. First, many studies focus exclusively on vertical traffic signs without addressing horizontal road markings within the same operational workflow. Second, many approaches are benchmark-oriented and are not directly designed for deployment in road-management applications. A similar fragmentation is observed for horizontal road markings, where the literature often focuses on standalone perception tasks, such as lane detection or segmentation. Representative works include SCNN [13], which models spatial relationships for lane detection, VPGNet [14], which jointly addresses lanes and road markings under challenging visual conditions, and LaneNet-style instance-based approaches [15] for end-to-end lane extraction. Segmentation-based approaches have also been investigated for road-marking detection, including Mask R-CNN-based instance segmentation models [16] and more recent encode–decode instance segmentation networks for lane-marking detection [17]. Recent reviews further confirm that deep-learning-based lane and road-marking detection is a mature and rapidly evolving research area, although it remains predominantly oriented toward autonomous-driving perception rather than integrated road-asset inventory generation [18]. These studies are highly relevant for road-scene understanding and autonomous-driving perception; however, they are generally not designed as unified road-asset inventory frameworks integrating both vertical traffic signs and horizontal road markings within the same low-cost acquisition and georeferencing workflow. A practical and reproducible framework for automated road-asset mapping based on low-cost vehicle-mounted action cameras is proposed. The methodology employs a dual-pipeline approach in which two specialized deep-learning models, one for vertical traffic signs and one for horizontal road markings, operate independently to account for the different geometry and visual characteristics of the two asset types. The outputs of the two pipelines are then unified through a spatial integration layer that combines visual detections with synchronized GPS data, transforming image-level detections into georeferenced map objects. Furthermore, a distance-aware frame extraction strategy is introduced to exploit vehicle trajectory information, reduce data redundancy, and improve spatial coverage compared with conventional fixed-rate sampling. This strategy supports the construction of a more spatially diverse dataset while limiting the inclusion of near-duplicate frames acquired over short travelled distances.

The remainder of the paper is organized as follows: Section 2 describes the experimental setup and data-acquisition system. Section 3 presents the dataset-construction strategy, including distance-aware sampling and class balancing. Section 4 details the adopted models and training procedures. Section 5 reports the experimental results for both vertical and horizontal assets and discusses the comparative analysis of the evaluated architectures.

## 2. Materials and Methods

The experimental infrastructure was designed to acquire high-quality visual and positioning data under real-world driving conditions, ensuring a representative characterization of different road scenarios. The data acquisition phase was based on an instrumented vehicle specifically equipped to operate as a mobile sensing platform for road infrastructure analysis. The vehicle was selected for its structural stability, which allowed the optical sensors to be installed with a consistent and vibration-resistant field of view across different operational conditions, including high-speed road sections and irregular urban pavements. The hardware architecture integrated into the vehicle consisted of a high-resolution optical sensing system capable of capturing the visual details required for the dual task of detecting distant vertical infrastructure and horizontal road markings. For this purpose, the vehicle was equipped with GoPro action cameras, selected for their high-resolution video recording capability, integrated GPS, portability, and straightforward workflow for mounting, configuration, and data acquisition. To collect data for both objectives, namely vertical sign detection and horizontal road marking detection, two action cameras were installed: one at the front of the vehicle and one at the rear. This configuration enabled simultaneous recordings from both viewpoints. Alternatively, the same survey can be performed using a single camera by repeating the acquisition twice, changing the mounting position, and reconstructing the vehicle trajectory using the integrated GPS. The cameras were configured to acquire videos at a native HD resolution of 1920 × 1080 pixels with a frame rate of 120 fps. This temporal and spatial resolution supports the object detection pipeline by ensuring that even small or distant signs, which may occupy only a limited portion of the frame, retain sufficient detail for accurate feature extraction and class identification. The embedded positioning system is based on a u-blox UBX-M8030 GNSS chip (u-blox AG, Thalwil, Switzerland), which supports concurrent multi-GNSS reception and provides a nominal horizontal position accuracy of 2.0 m CEP under the default operating mode. The GPS information recorded by the cameras was used to reconstruct the vehicle trajectory and associate the extracted frames with their corresponding spatial positions. This hardware configuration represents a complete operational pipeline designed to connect data acquisition, visual analysis, and georeferenced road-asset mapping. By using the vehicle as a standardized mobile sensing platform, the proposed framework provides a scalable solution for regional road safety assessments and proactive maintenance planning. The deep learning models were trained and evaluated on a workstation equipped with an Intel Core i7-13700F CPU and an NVIDIA GeForce RTX 4070 Ti SUPER GPU. All model inputs were resized to 640 × 640 pixels before training and inference. For the detection and subsequent classification of vertical signage, the forward-facing optical sensor was selected for its portability and suitability for high-dynamic-range acquisition conditions.

This aspect is particularly relevant for maintaining color consistency and edge definition in high-contrast environments, such as tunnels, shaded urban corridors, and road sections with rapidly changing illumination. A key component of the setup is the adoption of a standardized sensor geometry, based on the controlled height and pitch of the optical axis with respect to the road plane. Fixing these parameters improves the geometric consistency of the acquired imagery across the dataset, supporting the subsequent deep learning stages and reducing variability unrelated to the road assets themselves.

**Figure 1:**
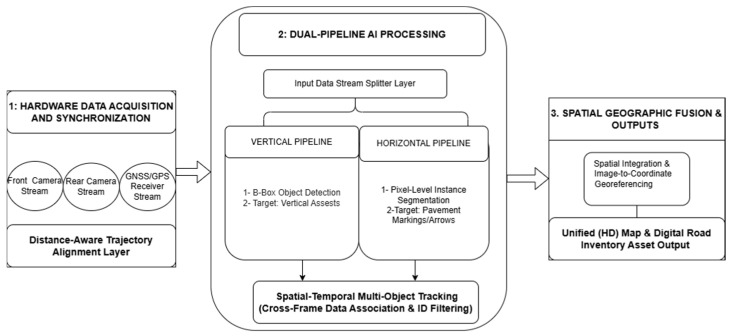
Overall workflow of the proposed road-asset mapping framework, including data acquisition, dual-pipeline processing, spatial integration, and final map generation.

The workflow illustrated in Figure 1 is organized into three main stages: data acquisition and synchronization, dual-pipeline processing, and geographic fusion with final map generation. In the first stage, the system acquires two complementary data streams: front and rear camera video streams, alongside GPS positioning data. The video stream provides the visual information required for road-asset recognition, while the GPS stream supplies the spatial reference associated with the vehicle trajectory. Since the two data streams are acquired at different native sampling rates and are not inherently aligned frame by frame, a distance-aware synchronization procedure is applied. Rather than relying exclusively on timestamps, this approach uses the travelled distance as a common reference to associate each video segment or frame with the corresponding geographic position. This step is essential to ensure spatial coherence between visual observations and positioning data, especially in mobile mapping applications where vehicle speed may vary during the survey. Once synchronized, the data enters the second stage, which is based on a dual-pipeline processing architecture designed to manage different classes of road assets separately. The first branch, referred to as the vertical asset pipeline, is devoted to the analysis of traffic signs and other upright roadside objects. In this case, the visual data are processed through a deep object detection and classification model, which identifies the presence of each asset in the scene, localizes it through bounding boxes, and assigns it to the corresponding semantic category. This pipeline is particularly suitable for discrete objects characterized by relatively compact and clearly distinguishable visual patterns. The second branch, referred to as the horizontal asset pipeline, addresses road markings and other painted or surface-level elements distributed on the pavement. These assets are processed using deep instance segmentation, which enables the identification and delineation of individual road-surface elements at the pixel level. This approach is particularly suitable for elongated, continuous, or irregular patterns, such as pedestrian crossings, stop lines, arrows, and other pavement markings, because it allows the shape and spatial extent of each detected element to be represented more accurately than with conventional bounding-box-based detection. The separation into two dedicated pipelines allows the system to exploit the specific characteristics of vertical and horizontal assets, improving both detection robustness and semantic interpretation. In the third stage, the outputs produced by the two vision pipelines are merged with the synchronized GPS information through a spatial integration and georeferencing module. At this stage, each detected or segmented road asset is assigned a geographic position derived from the vehicle coordinates and from its spatial relationship with the corresponding image frame. This fusion step transforms image-based detections into georeferenced entities that can be managed within a geographic framework. The final output of the workflow is a georeferenced road-asset map, which can support digital road inventory generation, high-definition map creation, and infrastructure monitoring applications. In practice, the system converts raw onboard acquisitions into structured spatial information, enabling the automatic representation of detected road elements on a map and supporting subsequent inspection, maintenance planning, and asset management tasks.

### 2.1. Vertical Road Signs Setup

To maximize the spatial coverage of the roadside environment, a wide field-of-view setting was adopted for the forward-facing camera. This configuration increases the probability of capturing vertical traffic signs and overhead structures over multiple consecutive frames, although it may introduce geometric distortions toward the edges of the image. To account for these effects, the detection model was trained using diverse samples, including signs located at different positions within the frame, in order to improve robustness against perspective variations and edge-of-frame distortions. The optical sensor was externally mounted on the vehicle at a height of 1.15 m. This elevation was selected to approximate the visual perspective of a human driver while maintaining a stable and unobstructed view of the roadside environment. The chosen mounting position serves two main purposes. First, it reduces visual occlusions caused by preceding or adjacent vehicles. Second, it provides a clear and consistent line of sight toward both pole-mounted traffic signs and elevated overhead gantries. This geometric consistency is particularly relevant for the tracking logic implemented during the inference phase, as it supports the association of detections across consecutive frames. The resulting acquisition configuration allows the vertical-sign pipeline to move beyond single-frame detection toward the construction of an automated inventory of vertical road assets. In this context, repeated detections of the same asset across multiple frames can be spatially associated and subsequently converted into a georeferenced road-inventory object.

**Figure 2:**
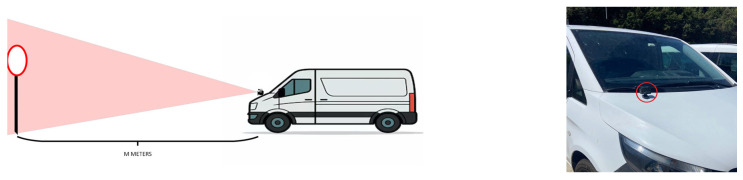
Example of the forward-facing acquisition geometry adopted for vertical traffic sign detection (**left**) and real-world mounting position of the front camera on the vehicle (marked with red circle) (**right**).

The geometric logic adopted for vertical signage acquisition is illustrated in Figure 2. The system is designed to maximize the detection window by adopting a long-range look-ahead perspective. As shown in Figure 2, the front-mounted camera monitors a longitudinal distance of approximately 13 m ahead of the vehicle. This spatial configuration provides the deep learning model with multiple observations of pole-mounted signs and overhead gantries as they progressively approach the vehicle. Capturing vertical assets from a distance allows the objects to be processed by the detection and classification pipeline before they exit the sensor field of view or become affected by short-range occlusions. This long-range orientation is therefore a key element of the tracking logic, supporting consistent frame extraction and repeated asset observation along the vehicle trajectory.

### 2.2. Horizontal Road Marking Acquisition Setup

The acquisition and mapping of horizontal road markings required a dedicated spatial configuration, since these elements are located on the pavement surface and are often characterized by elongated, irregular, or partially degraded geometries. For this task, the vehicle was equipped with a dedicated rear-mounted action camera, oriented toward the road surface behind the vehicle. The rear-facing configuration was selected to improve the visibility of pavement markings under real-world traffic conditions. In active road environments, preceding vehicles may partially or completely occlude the road surface in front of the survey vehicle. By positioning the camera at the rear, the acquisition system exploits the free road-surface area immediately behind the vehicle, reducing the probability of occlusions caused by other vehicles and allowing pavement markings to be captured more clearly. This configuration also contributes to improving the consistency of the acquired imagery. Since the camera records the pavement immediately after the vehicle has passed over it, the target area is less affected by frontal occlusions and short-range visual interference. The rear-mounted position can also help reduce some illumination variability associated with rapidly changing frontal shadows, although lighting conditions remain dependent on the surrounding environment, solar position, and road context. The internal electronic image stabilization of the action camera was used to improve image sharpness and reduce motion-related blur during acquisition. This aspect is particularly relevant for surface-level features such as lane arrows, pedestrian crossings, stop lines, edge lines, and other painted markings. The optical axis of the camera was configured with a downward pitch to focus on the road plane, producing a near top-down perspective of the pavement surface. This geometry reduces perspective distortion and supports the visual recognition of markings whose shape and proportions are important for semantic interpretation. The adopted rear-facing configuration is particularly suitable for polygonal annotation and instance segmentation. Unlike vertical signs, which can generally be represented by bounding boxes, horizontal markings often present extended, discontinuous, or irregular shapes. A downward-oriented view allows their actual geometry to be represented more accurately through segmentation masks, supporting both model training and subsequent automated cataloging.

**Figure 3:**
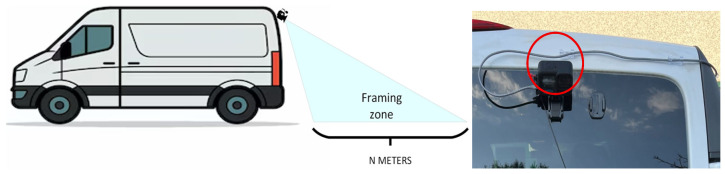
Example of the rear-facing acquisition geometry adopted for horizontal road marking detection and segmentation. The illustration (**left**) shows the camera orientation toward a dedicated pavement framing zone behind the vehicle, complemented by a photograph (**right**) displaying the actual camera placement (marked with red circle) on the vehicle’s rear.

The geometric configuration adopted for horizontal road marking acquisition is illustrated in Figure 3. The rear-mounted action camera was calibrated with a steep downward pitch to focus on a dedicated framing zone corresponding to an approximately 6.5 m longitudinal segment of the road surface behind the vehicle. This restricted acquisition window increases the pixel density on the pavement area of interest and reduces perspective distortion compared with wider or more oblique views. As a result, the deep learning model processes geometrically more consistent imagery of horizontal markings, which is essential for accurate segmentation, polygonal annotation, and georeferenced cataloging of pavement assets.

### 2.3. System Activation and Data Alignment

To ensure temporal and spatial consistency between the front-facing and rear-facing cameras, the data acquisition process was initiated through a centralized mobile application. This control interface enabled the simultaneous activation of both action cameras, ensuring that the two video streams were aligned from the beginning of the survey. This initial synchronization is particularly important because the two cameras acquire complementary views of the same road trajectory, with the front-facing camera dedicated to vertical road signs and the rear-facing camera dedicated to horizontal pavement markings. After data acquisition, a post-processing workflow was applied to reconstruct the survey trajectory and derive georeferenced image sequences. Telemetric metadata recorded by the action cameras, including GPS information, was extracted from the video files and converted into standardized GPX and CSV formats. These files provided the basis for reconstructing the vehicle trajectory and associating each video frame with the corresponding spatial position along the surveyed route. The extracted trajectory was then integrated with the camera calibration parameters and the known mounting positions of the sensors on the vehicle. This information was used to implement a distance-aware sampling strategy, in which video frames were extracted at fixed spatial intervals rather than at fixed temporal intervals. For example, in the horizontal road marking pipeline, frames were extracted every 6.5 m to match the longitudinal extent of the rear-facing pavement framing zone. This strategy reduces the redundancy typically associated with fixed-rate video sampling, where several consecutive frames may represent nearly identical road portions, especially at low vehicle speeds. At the same time, distance-aware sampling ensures more uniform spatial coverage of the road infrastructure regardless of speed variations during the survey. The resulting curated image sequence is therefore better suited for consistent spatial analysis, dataset construction, and subsequent georeferenced road-asset mapping.

**Figure 4:**
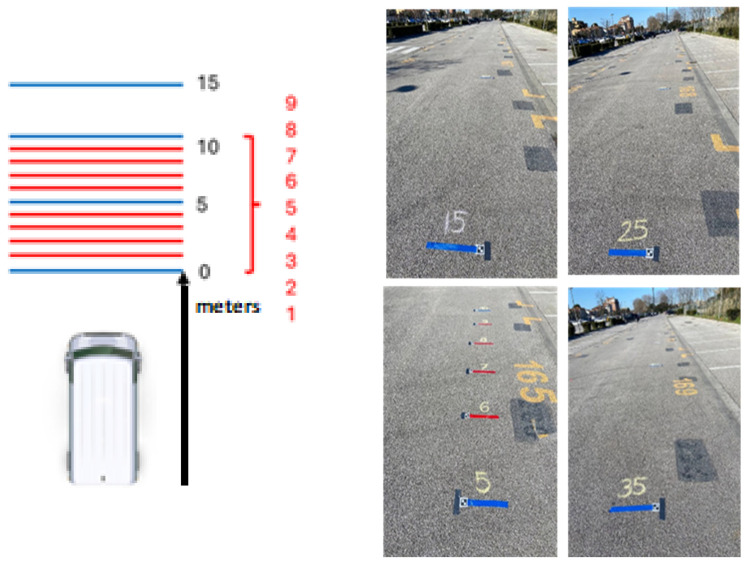
Validation of the distance-aware sampling strategy along a 50 m test trajectory. The schematic representation on the left shows the predefined spatial trigger points used for frame extraction: blue horizontal lines indicate the main distance reference positions, red lines and numbers indicate the consecutive extracted frames/checkpoints, and the black arrow represents the direction of travel and increasing distance from the vehicle. The image sequence on the right provides a visual verification based on pavement reference markers, with the numbers indicating the corresponding distance in meters.

As shown in Figure 4, the distance-aware sampling strategy was validated along a 50 m test trajectory using pavement reference markers as visual ground-truth points. The extracted frames correspond to the expected spatial positions, confirming the ability of the system to sample the video stream according to travelled distance rather than elapsed time. This validation supports the use of spatially triggered frame extraction for the construction of a consistent and non-redundant image dataset.

### 2.4. Quantitative Ablation of the Distance-Aware Sampling Strategy

To quantitatively assess the effectiveness of the distance-aware frame extraction strategy, an ablation analysis was performed by comparing the proposed spatial sampling method with a conventional fixed-time sampling approach. This ablation focuses on the sampling strategy itself, since the objective is to verify whether distance-aware extraction reduces spatial redundancy and improves the uniformity of road coverage under real driving conditions. The analysis was carried out separately for the two acquisition geometries adopted in the framework. For the horizontal road marking pipeline, three representative GPS trajectories were analyzed, including 35,736 GPS records, a total duration of 3573.299 s, and a travelled distance of 51,846.52 m. The average speeds ranged from 33.11 km/h to 61.24 km/h, covering both slower urban/suburban segments and more continuous extra-urban driving conditions. For this pipeline, the distance-aware strategy selected candidate frames every 6.5 m, consistently with the longitudinal extent of the rear-facing pavement framing zone. For the vertical traffic sign pipeline, the same ablation procedure was repeated using the spatial interval associated with the forward-facing acquisition geometry. Four representative GPS trajectories were analyzed, including 38,483 GPS records, a total duration of 2123.806 s, and a travelled distance of 33,037.29 m. The average speeds ranged from 37.24 km/h to 75.98 km/h. For this pipeline, the target spacing was set to 13 m, consistent with the forward-looking acquisition geometry used for vertical-sign detection. For a fair comparison, the fixed-time sampling strategy was configured separately for each trajectory to generate the same number of sampled positions as the corresponding distance-aware extraction. In both cases, the selected samples were associated with the nearest available GPS record, and the spatial distances between consecutive sampled positions were computed along the cumulative trajectory. Table 1 summarize the comparison that was based on indicators selected to quantify spatial redundancy and spatial coverage. The mean inter-sample spacing describes the average spatial distance between consecutive selected frames. The standard deviation and coefficient of variation in the spacing quantify the regularity of the sampling process. Short intervals, defined as shorter than half of the target spacing, indicate potentially redundant samples acquired over nearly the same road portion. Long intervals, defined as longer than 1.5 times the target spacing, indicate spatial gaps in the surveyed trajectory. Finally, the percentage of intervals within ±20% of the target spacing provides a direct measure of how consistently each method respects the desired spatial sampling interval.

Although the distance-aware and fixed-time strategies were configured to generate the same number of sampled positions, their spatial behavior was markedly different. For horizontal road markings, distance-aware sampling achieved a spacing standard deviation of 0.77 m, compared with 2.76 m for fixed-time sampling. Moreover, no short or long intervals were observed with distance-aware sampling, whereas fixed-time sampling produced 968 short intervals and 1020 long intervals. The percentage of intervals within ±20% of the target spacing was 89.14% for distance-aware sampling and only 35.39% for fixed-time sampling. The same trend was observed for the vertical traffic sign pipeline. Distance-aware sampling achieved a spacing standard deviation of 0.44 m, compared with 4.76 m for fixed-time sampling. No short or long intervals were observed with the proposed strategy, while fixed-time sampling produced 216 short intervals and 194 long intervals. In this case, all distance-aware intervals remained within ±20% of the target 13 m spacing, compared with 57.09% for fixed-time sampling. These results demonstrate that fixed-time sampling is strongly affected by vehicle speed variations. When the vehicle slows down, it produces spatially close samples that are likely to be redundant; when the vehicle accelerates, it produces larger spatial gaps along the surveyed route. Conversely, distance-aware sampling directly controls the travelled distance between consecutive selected frames, producing a more uniform spatial distribution. This quantitatively supports the use of the proposed strategy for reducing spatial redundancy and improving the representativeness of the selected frames during dataset construction.

## 3. Dataset Creation

The development of a training dataset for road infrastructure detection requires a data-curation strategy capable of ensuring environmental variability, spatial representativeness, annotation consistency, and independence among data subsets. In mobile video surveys, conventional time-based frame extraction may generate many redundant images, especially when the vehicle is stopped or moving slowly, since consecutive frames often represent nearly identical road portions and backgrounds. To reduce this redundancy, a distance-aware sampling strategy was adopted. Frames were extracted at fixed spatial intervals using the GPS trajectory of the survey vehicle, rather than at fixed temporal intervals. This ensured that each selected image corresponded to a different position along the route, improving spatial diversity and exposing the models to variations in background, viewpoint, scale, illumination, and partial occlusion. The selected interval also supported multi-view observation of the same asset categories as the vehicle approached, passed, or moved away from them. The datasets were split at the image level using frames extracted from different video sequences and road routes. Training, validation, and test subsets were kept mutually independent, meaning that the same extracted image was never included in more than one subset. The split preserved the class distribution across subsets while ensuring independent image-level test sets for final evaluation. Overall, the adopted strategy reduced near-duplicate samples, improved spatial coverage, and supported a more robust assessment of the proposed models.

### 3.1. Vertical Signage Dataset

The vertical signage dataset was developed from high-resolution frames acquired by the forward-facing action camera mounted on the survey vehicle. Since vertical traffic signs are generally characterized by standardized and rigid geometric shapes, such as circles, triangles, rectangles, octagons, and inverted triangles, the dataset was annotated using rectangular bounding boxes. The annotation process was performed using LabelImg, an open-source graphical image annotation tool. Each bounding box was drawn to include the complete sign face while minimizing the surrounding background, providing clear ground-truth localization information for model training. A standardized preprocessing workflow was applied to prepare the dataset for deep learning. All selected frames were resized to a uniform resolution of 640 × 640 pixels. This input size was selected to match the requirements of the adopted YOLO-based detection architecture, while balancing computational efficiency and the preservation of spatial details required for detecting small or distant signs. In addition to resizing, a vertical cropping strategy was applied to the original wide-angle images, in which the images were vertically cut by removing the left and right regions. This preserves the original pixel information within the retained area, avoiding the loss of detail associated with direct resizing. The distance-aware extraction process allowed the dataset to include road signs acquired across urban, suburban, and extra-urban contexts.

This variability is important for exposing the model to different backgrounds, lighting conditions, road geometries, and traffic scenarios. The vertical signage dataset was organized into ten primary classes representing the main regulatory and safety-related categories considered in this study: danger signs, prohibition signs, priority signs, mandatory signs, stop signs, give-way signs, no-parking/no-stopping signs, no-entry signs, pedestrian-crossing signs, and traffic lights.

**Figure 5:**
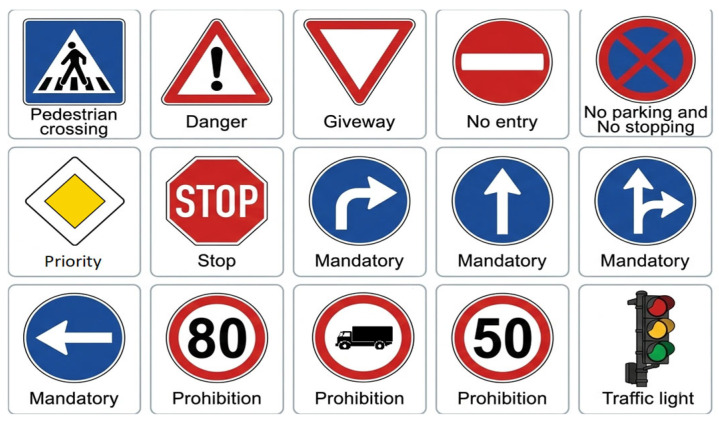
Examples of the vertical traffic sign classes included in the dataset.

As shown in Figure 5, the dataset includes a diverse set of vertical road signs categorized according to their regulatory and safety functions. Priority and mandatory signs, such as stop and give-way signs, are essential for right-of-way management and are characterized by distinctive geometric shapes. Prohibition and entry-restriction signs, including no-entry and speed-limit signs, provide relevant information for digital road inventories and navigation-related applications. The dataset also includes warning and control elements, such as danger signs, pedestrian-crossing signs, and traffic lights, which are important for both conventional road management and intelligent transportation systems. The resulting vertical signage dataset provides a structured representation of the main traffic-sign categories encountered during the survey campaign. The combination of distance-aware sampling, controlled preprocessing, and class-based annotation supports the construction of a spatially diverse dataset, reducing the influence of redundant backgrounds and improving the generalization capability of the detection model. The final distribution of annotated instances for each class is reported below.

#### Synthetic Data Generation and Class Balancing

Class imbalance is a common issue in road-sign datasets, since some categories occur frequently in real-world driving scenarios, whereas others are observed only in specific road contexts. This imbalance can affect model training by favoring the recognition of the most represented classes and reducing detection robustness for less frequent but safety-relevant signs. To mitigate this effect, a synthetic data generation procedure was introduced to complement the real-world images and improve the representation of underrepresented classes. The synthetic data generation workflow was based on the combination of real road backgrounds and digital traffic-sign templates. Synthetic data generation and augmentation have been widely investigated in traffic-sign recognition and detection, including GAN-based generation strategies and approaches based on the insertion of synthetic traffic signs into road-scene images [19,20]. These studies show that synthetic samples can increase visual diversity and improve the representation of underrepresented classes, provided that they are used to complement rather than replace real-world data. First, negative background images were extracted from the acquired video frames by selecting scenes that did not contain visible traffic signs. These images provided realistic roadside contexts, including urban, suburban, and extra-urban environments. Then, high-quality digital sign icons were superimposed onto the selected backgrounds. The synthetic signs were randomly transformed in terms of scale, position, and perspective in order to simulate different viewing distances, acquisition angles, and positions within the camera field of view.

This procedure allowed the generation of additional training samples while preserving realistic background variability. The objective was not to replace real-world annotations, but to supplement the dataset with controlled examples of underrepresented classes. This hybrid strategy is consistent with previous work highlighting both the potential and the limitations of synthetic traffic-sign repositories, particularly the need to bridge the domain gap between real and generated samples [21]. In this way, the model was exposed to a more balanced set of visual conditions, reducing the risk of class-specific bias during training and improving generalization for less frequent road-sign categories.

**Figure 6:**
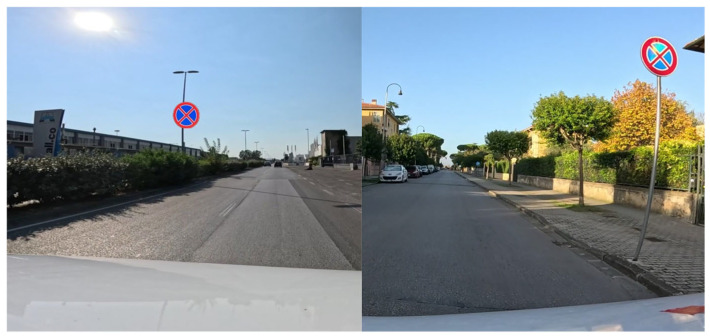
Comparison between synthetic (**left**) and real vertical traffic sign (**right**) samples. The synthetic example was generated by superimposing a digital sign template onto a real road background, while the real example was extracted from the acquired survey footage.

As shown in Figure 6, the synthetic samples reproduce the main visual characteristics of real traffic signs while maintaining realistic roadside backgrounds. This hybrid strategy supports class balancing and increases the variability of the training dataset, particularly for categories that are less frequently observed during ordinary road surveys. The final distribution of each class instance is presented in Table 2 below.

In the final vertical traffic sign dataset, synthetic images accounted for approximately 38.6% of the final training set. Specifically, 4303 synthetic images were used out of 11,161 total training images, corresponding to a ratio of approximately 61.4% real-world samples and 38.6% synthetically generated samples. Synthetic samples were mainly introduced to improve the representation of underrepresented, safety-relevant classes and to expose the model to additional variations in scale, position, perspective, and image location. This controlled injection of synthetic data was therefore used as a complementary class-balancing strategy rather than as a replacement for real-world observations.

### 3.2. Horizontal Road Marking Dataset

The horizontal road marking dataset was developed from frames acquired by the rear-facing action camera mounted on the survey vehicle. Unlike vertical traffic signs, horizontal markings are located on the pavement surface and are often characterized by elongated, irregular, or partially degraded shapes. For this reason, the dataset construction required a dedicated annotation strategy based on polygonal segmentation rather than conventional bounding boxes. An initial evaluation was conducted using publicly available datasets from platforms such as Roboflow [22] and Kaggle [23]. These datasets provided a useful baseline for preliminary testing, but several limitations were identified, including heterogeneous annotation formats, differences in road-marking styles, and limited consistency with the acquisition geometry adopted in this study. Consequently, a custom dataset was created from the vehicle-mounted rear-facing camera acquisitions, ensuring full coherence between the training data and the operational sensing configuration. The class taxonomy was defined through an iterative refinement process. Initial experiments considered several common pavement markings, including give-way markings, pedestrian crossings, directional arrows, stop markings, and lane-related lines. However, during preliminary model evaluation, solid and overtaking lines showed strong geometric similarity and produced frequent ambiguities in the predictions. For this reason, these line-based classes were excluded from the final operational dataset, allowing the model to focus on more visually distinctive and semantically relevant categories. The final horizontal road marking dataset included nine classes: give-way markings, left arrows, right arrows, stop markings, straight arrows, straight-left arrows, straight-right arrows, triangular give-way markings, and pedestrian crossings. Frames were extracted at fixed 6.5 m spatial intervals from the recorded videos, following the distance-aware sampling strategy described in Section 2.3. This procedure ensured uniform spatial coverage across urban and extra-urban road environments while reducing redundancy among consecutive frames. All selected images were resized to a uniform resolution of 640 × 640 pixels before annotation and model training.

**Figure 7:**
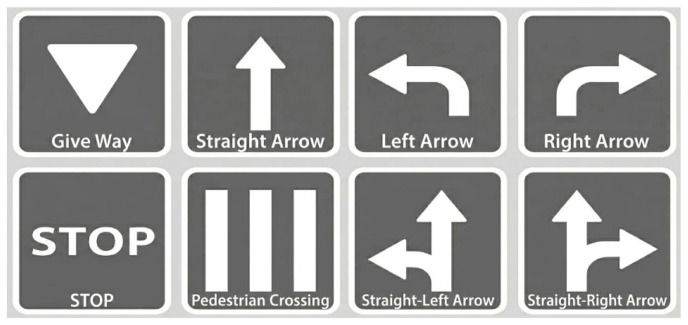
Examples of horizontal road marking classes included in the dataset.

As shown in Figure 7, the dataset includes pavement markings with different geometric structures and traffic functions. Regulatory markings, such as stop and give-way symbols, provide priority information, while pedestrian crossings and directional arrows support traffic organization and road-user guidance. The exclusion of ambiguous line-based categories reduced class overlap and improved the semantic consistency of the dataset.

#### Polygonal Annotation and Class Balancing for Horizontal Road Markings

Horizontal road markings were annotated using polygonal segmentation instead of rectangular bounding boxes. This choice was motivated by the geometric characteristics of pavement markings, which are often elongated, irregular, partially worn, or affected by perspective distortion.

In these cases, bounding boxes may include large portions of irrelevant asphalt background and may not accurately represent the actual shape of the asset. Polygonal segmentation allows the visible contour of each marking to be traced at the pixel level, producing a mask that more closely follows the real geometry of the road asset. This approach is particularly relevant for directional arrows, triangular give-way markings, pedestrian crossings, and stop markings, whose semantic interpretation depends on shape, orientation, and spatial extent. By preserving the morphology of the marking, segmentation masks provide more informative ground-truth data for model training than bounding boxes alone. Synthetic data generation was then used to improve class balance in the horizontal road marking dataset. Some pavement-marking classes are more frequently encountered during ordinary road surveys, whereas others appear only in specific traffic configurations. This uneven distribution may affect the training process by favoring the most represented classes and reducing robustness for less frequent markings.

The synthetic generation procedure was based on the combination of real pavement backgrounds and digital marking templates. Negative background images were extracted from the rear-facing camera footage by selecting frames without visible road markings. Digital templates of the underrepresented markings were then superimposed onto these backgrounds. This procedure follows the same rationale adopted in synthetic traffic-sign augmentation, where controlled insertion of visual templates into real road backgrounds has been used to increase class diversity while preserving realistic environmental context [20,21]. The synthetic samples were generated by varying scale, position, orientation, and perspective in order to reproduce different acquisition geometries and viewing conditions. This procedure increased the number and variability of training samples for less represented classes while preserving realistic pavement textures and illumination conditions. The objective was not to replace real annotated images, but to complement them with controlled examples useful for balancing the dataset and improving model generalization.

**Figure 8:**
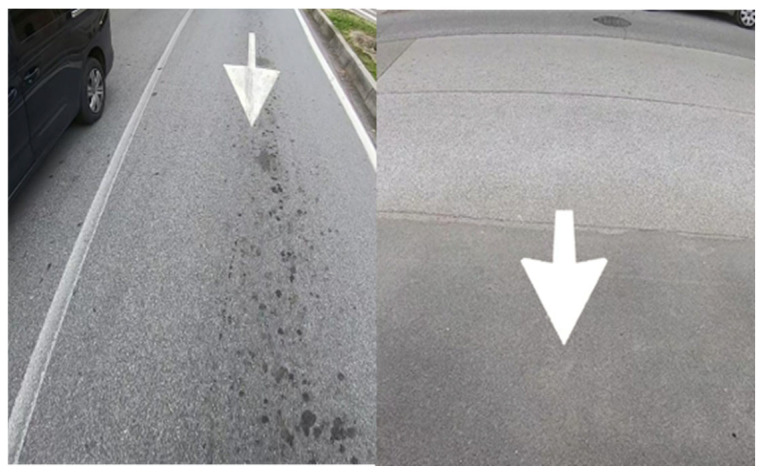
Comparison between real (**left**) and synthetic (**right**) horizontal road marking samples. The synthetic sample was generated by superimposing a digital marking template onto a real pavement background while varying scale, orientation, and perspective.

As shown in Figure 8, the synthetic samples reproduce realistic pavement contexts and geometrical variations in horizontal markings. This hybrid strategy supports class balancing and increases the variability of the training dataset, particularly for classes that are less frequently observed in real-world acquisitions. The final distribution of annotated instances for each horizontal road marking class is reported in Table 3. The dataset was divided into training, validation, and test subsets while preserving the class structure across the three partitions.

In the final horizontal road marking dataset, synthetic images accounted for approximately 20.3% of the complete image set. The dataset included 8166 images and 8353 annotated instances, with 1656 images generated synthetically and 6510 images extracted from real survey footage. This corresponds to a ratio of approximately 79.7% real-world images and 20.3% synthetic images. Synthetic samples were selectively introduced to complement real observations and improve the balance of less frequent pavement-marking classes, particularly those associated with specific traffic configurations or directional information.

## 4. Deep Learning Models and Training Setup

The proposed framework employs a dual-pipeline approach tailored to the distinct geometric properties of road assets. Vertical traffic signs are treated as a multi-class object detection problem, utilizing rectangular bounding boxes for these discrete, compact objects. Conversely, horizontal road markings are addressed via instance segmentation to manage elongated, irregular shapes and perspective distortions through pixel-level polygonal masks.

Three architectural families were evaluated to assess the robustness of the proposed framework:

- YOLO11m [24]: an efficient one-stage architecture used for vertical traffic sign detection. Its segmentation variant, YOLO11m-seg, was adopted for horizontal road marking instance segmentation.
- RT-DETR [25]: a transformer-based detector selected for its ability to exploit global image context.
- Faster R-CNN [26]: a consolidated two-stage baseline based on region proposals, used as a reference architecture for object localization.

For the vertical pipeline, all models were evaluated using bounding-box annotations. In the horizontal pipeline, YOLO11m-seg served as the primary segmentation engine, while RT-DETR and Faster R-CNN provided box-based detection baselines to assess the added value of mask-based representation. All training inputs were standardized to a resolution of 640 × 640 pixels. This distinction between the vertical and horizontal pipelines ensures robust localization for vertical signs and precise morphological representation for surface-level pavement markings. The inference speed of the selected YOLO11m-seg model was approximately 8 ms per image on the adopted workstation, corresponding to about 125 frames per second at 640 × 640 pixel input resolution. This distinction ensures robust localization for vertical signs and precise morphological representation for surface-level pavement markings.

### Training Configuration and Evaluation Metrics

The datasets were divided into independent training, validation, and test subsets. The validation sets were used during model development and parameter selection, while the test sets were kept separate and used only for the final performance assessment. The training and evaluation configuration adopted for the two pipelines is summarized in Table 4.

Precision and recall were used to evaluate the balance between false detections and missed detections, while the F1-score was adopted as the main summary metric because it combines both indicators into a single measure. The mAP50 metric was used to evaluate detection performance at an Intersection over Union threshold of 0.50, whereas mAP50-95 provided a stricter assessment by averaging performance over multiple IoU thresholds. For the horizontal road-marking pipeline, the comparison among YOLO11m-seg, RT-DETR, and Faster R-CNN was performed using the F1-score, which provided a common metric for all evaluated architectures. This choice was necessary because RT-DETR and Faster R-CNN were used as detection-only baselines and do not produce segmentation masks. For the selected YOLO11m-seg model, both box-level and mask-level metrics were additionally computed. In particular, mask mAP50 and mask mAP50-95 were used to assess the quality of the predicted segmentation masks on the independent test set. Therefore, the cross-model comparison was based on F1-score, while the final YOLO11m-seg evaluation included true mask-level segmentation metrics. To verify the stability of the training process, the learning curves of the final selected models were analyzed for both recognition tasks. In particular, the convergence behavior of YOLO11m was evaluated for vertical traffic sign detection, while YOLO11m-seg was analyzed for horizontal road-marking segmentation. The training and validation curves provided useful information on the optimization process and allowed possible overfitting or unstable learning behavior to be identified.

**Figure 9:**
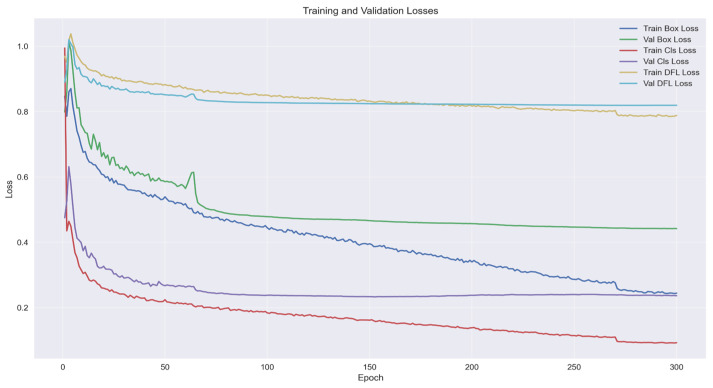
Training and validation loss curves of the YOLO11m model selected for vertical traffic sign detection. The plot reports box loss, classification loss, and distribution focal loss (DFL) for both training and validation over 300 epochs.

As shown in Figure 9, the YOLO11m model exhibited a stable convergence behavior over the 300 training epochs. All training losses progressively decreased, indicating that the model effectively learned the localization and classification features required for vertical traffic sign detection. The validation losses followed the same general trend, with a rapid decrease during the initial epochs followed by a gradual stabilization. The box loss decreased consistently for both training and validation, confirming an improvement in object localization during training. The classification loss also showed a clear reduction, indicating that the model progressively improved its ability to distinguish among the different traffic sign classes. The DFL curves remained higher than the other loss components but showed a stable and slowly decreasing trend, suggesting consistent refinement of bounding-box localization. Overall, the absence of a clear divergence between training and validation losses indicates that the model did not show evident overfitting. The convergence pattern supports the selection of YOLO11m as the final model for the vertical traffic sign detection pipeline.

**Figure 10:**
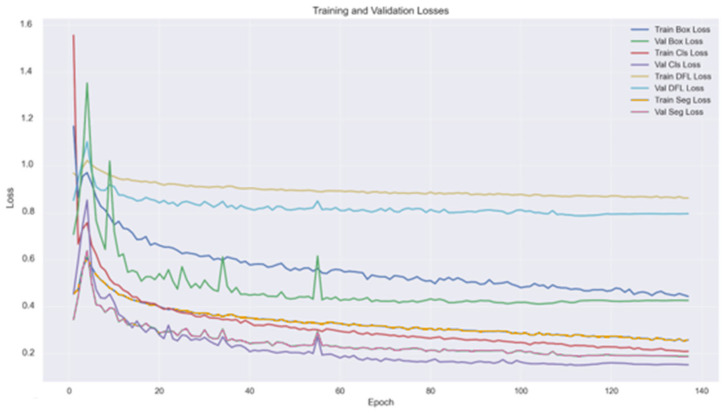
Training and validation loss curves of the YOLO11m-seg model selected for horizontal road marking segmentation. The plot reports box loss, classification loss, distribution focal loss (DFL), and segmentation loss for both training and validation over the training epochs.

As shown in Figure 10, the YOLO11m-seg model exhibited a stable convergence behavior during training. After an initial transient phase characterized by higher variability in the first epochs, the loss curves progressively decreased and then approached a stable trend. This behavior indicates that the model gradually learned both the localization and segmentation features required for horizontal road marking recognition. The box loss decreased for both training and validation, confirming an improvement in object localization. The classification loss also showed a clear reduction, indicating improved discrimination among the different pavement marking classes. The segmentation loss progressively decreased and stabilized, suggesting that the model learned to delineate the pixel-level shape of horizontal markings with increasing consistency. The DFL curves remained higher than the other components, as also observed for the vertical model, but followed a stable decreasing trend. Overall, the validation curves remained consistent with the training trends, without a marked divergence between the two sets. This indicates that the model did not show evident overfitting and supports the selection of YOLO11m-seg as the final model for the horizontal road marking segmentation pipeline.

To justify the selection of YOLO11m-seg for horizontal marking instance segmentation, its architectural trade-offs were evaluated against alternative mainstream frameworks:

- Architectural Constraints of Alternatives: Two-stage networks (e.g., Mask R-CNN) and transformer-based models can provide accurate segmentation in offline environments, but they generally involve higher computational complexity and memory requirements. In low-cost vehicle-integrated workflows, this may reduce processing throughput and increase the risk of missed observations when operating on dense video streams.
- Optimization and Structural Alignment: Pavement markings feature highly specific, elongated, and continuous geometries (e.g., directional arrows). YOLO11m-seg utilizes advanced single-stage cross-stage spatial attention modules that capture these long, narrow pixel structures effectively without the massive VRAM overhead of vision transformers.

As demonstrated by the empirical results, YOLO11m-seg achieved a global F1-score of 0.96 and a mask mAP50 of 0.98. In addition, the selected model reached an inference time of approximately 8 ms per image on the adopted workstation, corresponding to about 125 frames per second at 640 × 640 pixel input resolution. These results indicate that YOLO11m-seg provides a favourable balance between recognition accuracy, mask-level representation, and computational efficiency. For this reason, it was selected as the reference model for the horizontal road-marking pipeline within the proposed low-cost workflow.

## 5. Results

The performance of the proposed framework was evaluated separately for the two recognition tasks: vertical traffic sign detection and horizontal road marking segmentation. For each task, the tested architectures were first compared using global performance metrics. Then, a class-wise analysis was carried out for the best-performing model in order to identify the strengths and limitations of the final operational pipeline.

### 5.1. Vertical Traffic Sign Detection Results

The vertical traffic sign detection task was evaluated by comparing YOLO11m, RT-DETR, and Faster R-CNN on the independent test set. The comparison was performed using the main global metrics introduced in Section 4, namely precision, recall, mAP50, mAP50-95, and F1-score.

**Figure 11:**
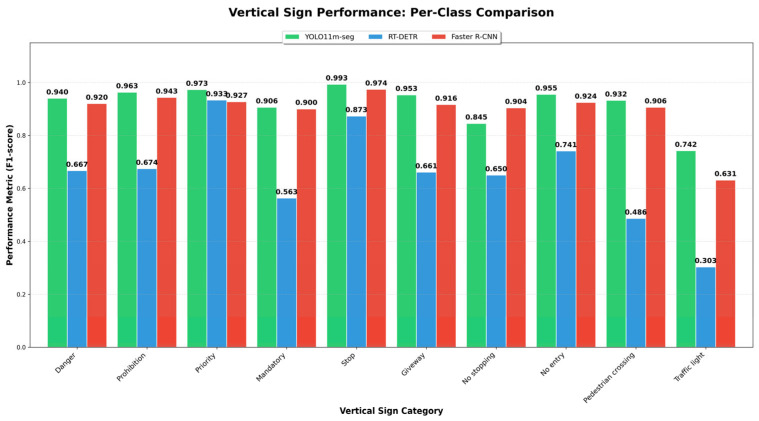
Comparison of test-set performance obtained by YOLO11m, RT-DETR, and Faster R-CNN for vertical traffic sign detection.

The comparative performance of the three evaluated architectures is illustrated in Figure 11. YOLO11m achieved the highest overall performance, reaching an average F1-score of 0.9202 and a mAP_50_ of 0.947. While Faster R-CNN maintained highly competitive results with an F1-score of 0.8945, its performance remained approximately 2.8% lower than the YOLO-based architecture. In contrast, RT-DETR exhibited significantly higher performance variability, resulting in a cumulative average F1-score of 0.6551. This represents a performance gap of approximately 28.8% compared to the leading model, primarily due to increased sensitivity and lower precision in visually complex or small-scale categories. These findings support the selection of YOLO11m as the reference model for the vertical asset pipeline.

Following the architectural comparison, a detailed class-wise analysis was conducted for the YOLO11m model to evaluate its behavior across individual vertical sign categories. The metrics derived from the testing phase, including precision, recall, and specific F1-scores per class, are reported in Table 5.

The YOLO11m model achieved an overall precision of 0.96, recall of 0.88, mAP50 of 0.94, mAP50-95 of 0.88, and F1-score of 0.92. These results indicate strong overall detection performance, with particularly high scores for visually distinctive classes such as Danger, Prohibition, Priority, Stop, and No-entry signs. In particular, the Stop class achieved the highest performance, with an F1-score of 0.99, confirming the robustness of the model for signs characterized by distinctive geometry and high visual contrast. Danger and Prohibition signs also showed high performance, with F1-scores of 0.94 and 0.96, respectively. Traffic lights achieved an F1-score of 0.74. Unlike standard roadside signs, which are generally located close to the driver’s eye level, traffic lights are often mounted at a greater height on poles or overhead gantries. Considering the camera mounting height of 1.15 m, these targets may therefore be affected by steep vertical perspective compression and reduced apparent size in the image. In addition, traffic lights present high visual variability because only one small, illuminated element may be active at a given time within a dark enclosure, increasing the risk of missed detections or confusion with complex urban backgrounds. The detection of traffic lights is known to be particularly challenging due to their small apparent size and visual variability in urban environments, as documented in previous studies [27].

The No-parking/No-stopping class achieved high precision but lower recall, resulting in an F1-score of 0.84. This indicates that detections were generally reliable when produced, but some instances were missed by the model. This behavior can be partly explained by the lower internal contrast of these signs compared with other prohibition signs. While standard prohibition signs typically combine black symbols, white backgrounds, and red borders, No-parking/No-stopping signs include a dark blue background crossed by red bars. From greater distances, and especially under harsh sunlight, shadows, or motion blur, these low-reflectance chromatic components may visually blend together, reducing the separability of the internal pattern and making the sign appear as a generic dark circular object within urban clutter.

### 5.2. Horizontal Road Marking Results

The horizontal road marking task was evaluated by comparing YOLO11m-seg, RT-DETR, and Faster R-CNN on the independent test set. The comparison was performed using the same global metrics adopted for the vertical task.

**Figure 12:**
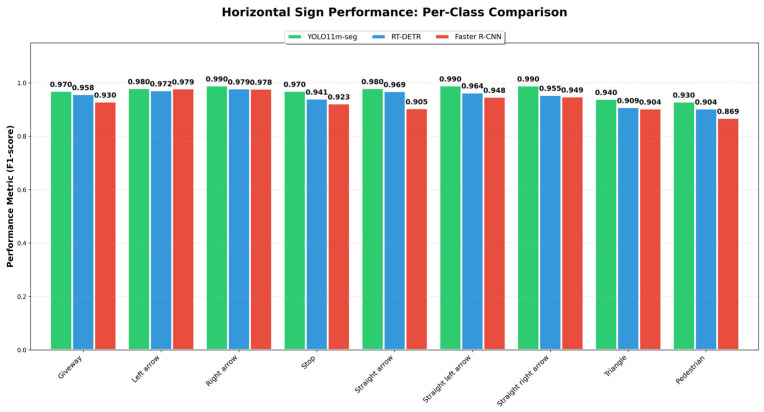
Comparison of test-set performance obtained by YOLO11m-seg, RT-DETR, and Faster R-CNN for horizontal road marking recognition.

Figure 12 compares the horizontal road-marking models using the F1-score, which was selected as the common metric across YOLO11m-seg, RT-DETR, and Faster R-CNN. This comparison shows that YOLO11m-seg achieved the best overall performance, with an F1-score of 0.96, followed by RT-DETR and Faster R-CNN. Since RT-DETR and Faster R-CNN were used as detection-only baselines and do not produce segmentation masks, mask-level metrics were computed only for the final YOLO11m-seg model. This additional evaluation included mask mAP50 and mask mAP50-95, allowing the segmentation quality of the selected model to be assessed directly. The selection of YOLO11m-seg as the reference model for the horizontal asset pipeline is therefore supported both by its superior F1-score in the cross-model comparison and by its ability to provide mask-based outputs that preserve the spatial extent and shape of pavement markings. After the model comparison, a class-wise analysis was conducted for YOLO11m-seg to evaluate its behavior across the individual horizontal marking categories. The corresponding results are reported in Table 6.

The YOLO11m-seg model achieved an overall precision of 0.98, recall of 0.96, mask mAP50 of 0.98, mask mAP50-95 of 0.91, and F1-score of 0.96. These results confirm the effectiveness of the segmentation-based approach for horizontal pavement assets. The best results were obtained for directional arrows, which are characterized by well-defined geometries and distinctive shapes. Right arrows, straight-left arrows, and straight-right arrows reached F1-scores close to 0.99, indicating strong discrimination among different directional markings. Lower scores were observed for pedestrian crossings and triangular give-way markings. These classes are more affected by pavement texture, surface wear, partial occlusion, and background similarity. In particular, the lower mAP50-95 values suggest that, although the model was generally able to detect these markings, the precise localization or segmentation of their full spatial extent was more challenging.

The effectiveness of the hybrid real/synthetic data strategy is also reflected in the final class-wise performance. In real road environments, vertical signs and horizontal markings naturally follow a long-tail distribution, in which some safety-relevant categories occur much less frequently than others. Without class-balancing strategies, models trained on highly unbalanced datasets may achieve good global performance while neglecting rare classes, leading to low recall and F1-scores for those categories. In this study, rare or safety-critical vertical sign classes, such as Stop and Give-way, achieved F1-scores of 0.99 and 0.95, respectively. Similarly, less frequent horizontal marking classes, including Stop marking, Give-way marking, Straight-left arrow, and Straight-right arrow, achieved F1-scores ranging from 0.97 to 0.99. Although a dedicated ablation study comparing real-only and real-plus-synthetic training was not performed, these results provide quantitative evidence that the controlled use of synthetic data supported robust performance for long-tail categories without reducing the overall accuracy of the framework.

The obtained results are consistent with recent studies on automated road-marking inspection using computer vision and deep learning. For example, street-view-based road-marking inspection systems have demonstrated the suitability of YOLO-based approaches for practical pavement-asset monitoring tasks [28]. Similarly, recent YOLO-based instance segmentation studies have reported high detection and segmentation performance for road-marking signs, confirming the relevance of mask-based architectures for representing pavement-marking geometry [29]. Compared with these task-specific studies, the present framework extends the analysis by integrating horizontal marking segmentation, vertical traffic sign detection, and GPS-based georeferenced mapping within a unified low-cost workflow.

### 5.3. Georeferenced Road-Asset Mapping Output

The final stage of the proposed framework consists of transforming image-level detections into georeferenced road-asset entities. The detections produced by the vertical and horizontal pipelines were associated with the synchronized GPS trajectory and represented in a cartographic environment. In this way, each detected asset was linked to a spatial position and to the corresponding metadata, including the source frame, asset category, and predicted class.

**Figure 13:**
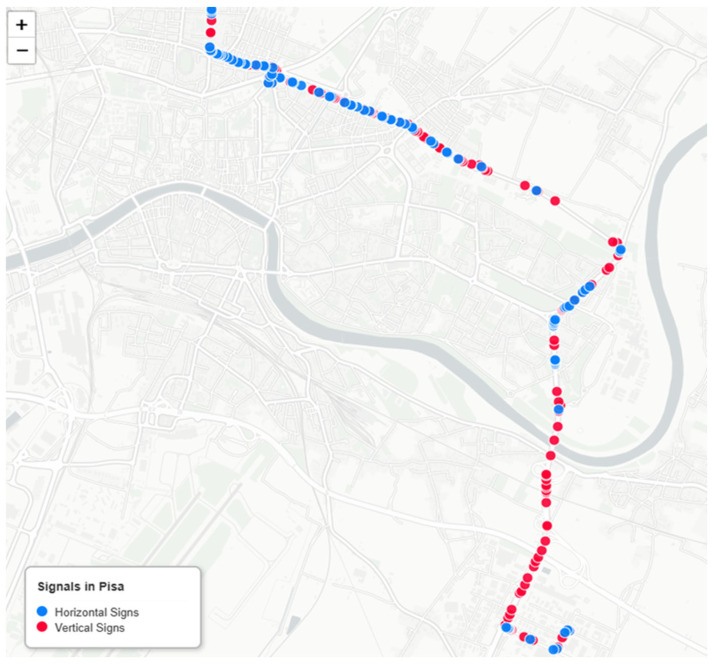
Georeferenced map of the detected road assets along the surveyed route. Blue points indicate horizontal road markings, while red points indicate vertical traffic signs.

As shown in Figure 13, the resulting map provides a spatial inventory of the detected road assets along the surveyed trajectory. The representation distinguishes between vertical and horizontal assets, allowing the distribution of different infrastructure elements to be inspected directly in geographic space. This output demonstrates that the proposed pipeline is not limited to image-level recognition, but can generate structured spatial information suitable for road inventory updating and maintenance planning.

**Figure 14:**
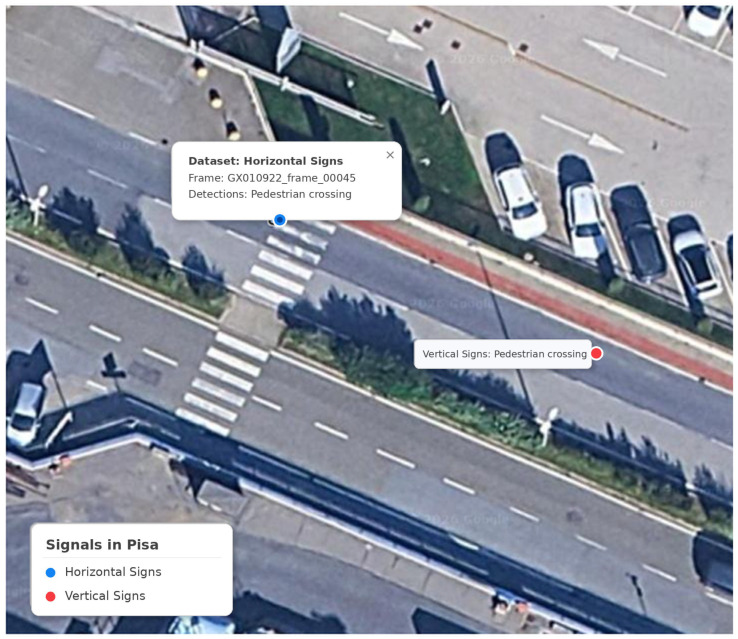
Example of a georeferenced road-asset detection with associated metadata. The pop-up links the mapped point to the source frame, dataset type, and detected class.

Figure 14 provides an example of the metadata associated with a mapped asset. Each point remains linked to the original visual detection, ensuring traceability between the cartographic output and the image-based recognition process. This feature is relevant for practical road-management applications, since detected elements can be inspected, verified, and updated within a georeferenced digital inventory.

It is worth noting that the georeferencing accuracy of the proposed framework is primarily constrained by two factors: the positional uncertainty of the consumer-grade GPS receiver embedded in the action cameras and the spatial coverage of the imaging pipeline. Previous studies on terrestrial mobile platforms have shown that GPS-based positioning accuracy can vary substantially depending on satellite geometry, multipath propagation, and the degree of integration with inertial sensors [30]. In the present low-cost configuration, the expected positional uncertainty is therefore on the order of several metres and propagates directly to the final road-asset map. This level of accuracy is consistent with the intended use of the framework for road inventory and maintenance planning, while remaining below the requirements of applications demanding lane-level or sub-meter localization accuracy. More advanced globally referenced mapping approaches based on standard GPS and multi-session processing have shown that substantially higher positional accuracy can be achieved under dedicated processing strategies [31]. Future developments will therefore explore the integration of improved GPS processing, differential corrections, or RTK positioning to enhance the absolute accuracy of the georeferenced output. The comparison with other low-cost mobile mapping systems for traffic sign localization further confirms that positional accuracy remains a central challenge for scalable and affordable road-asset mapping workflows [6].

### 5.4. Quantitative Evaluation of Georeferencing Accuracy

To quantitatively evaluate the georeferencing accuracy of the proposed low-cost mapping workflow, a manual validation was performed on 257 mapped assets, including 136 vertical traffic signs and 121 horizontal road markings distributed across urban, extra-urban, and motorway contexts. For each asset, the position assigned by the vision–GPS pipeline was compared with a manually verified reference position obtained through visual inspection of the source frames and georeferenced cartographic information. The positional error was computed as the planimetric distance between the automatically assigned coordinate and the reference coordinate. This validation focused on the final mapped-asset position, since the overall error depends not only on the consumer-grade GPS uncertainty, but also on the acquisition geometry and on the relationship between the vehicle-mounted camera and the detected asset. Different accuracy levels were observed for the two asset types. Horizontal road markings showed more controlled georeferencing conditions, as they are located on the road plane and acquired within a fixed rear-facing framing zone of approximately 6.5 m. Under stable reception conditions, the trajectory showed more regular positioning behaviour, while the final planimetric error for horizontal markings ranged from 0.45 m to 5.73 m, with an average value of 1.67 m. Conversely, vertical traffic signs showed higher variability because they are roadside objects observed from a moving vehicle and, in the current implementation, their position is associated with the vehicle’s GPS coordinate at the selected detection frame. Since the procedure does not explicitly model the lane of travel, lateral offset, side-of-road position, viewing distance, or road width, the resulting planimetric error ranged from 0.86 m to 18.03 m, with an average value of 4.71 m. These results indicate that the proposed workflow provides road-inventory-level georeferencing accuracy, suitable for asset inventory generation, maintenance planning, and preliminary infrastructure inspection, but not for lane-level or sub-meter localization. Future developments will focus on reducing the positional uncertainty of vertical assets through explicit camera-to-object geometric modelling, side-of-road estimation, lane-aware map matching, improved tracking of repeated detections, and the integration of higher-accuracy positioning technologies such as differential GPS or RTK-GPS.

### 5.5. Independent Operational Validation on Heterogeneous Road Types

To further assess the operational applicability of the proposed framework beyond the data used for model development, an additional independent road survey was carried out over approximately 70 km of real road network. This validation was performed using data not included in the training, validation, or test sets adopted for the deep learning models. The additional survey included different road typologies, namely urban roads, extra-urban roads, and motorway sections. These contexts differ in terms of vehicle speed, road width, lane configuration, sign placement, traffic conditions, visual background complexity, and pavement-marking geometry. Therefore, the survey provided a more heterogeneous operational scenario than a single local urban route. Across the independent validation survey, the framework detected 463 vertical traffic signs and 211 horizontal road markings. These detections were used to verify the ability of the complete vision–GPS workflow to operate on road sections that were not part of the model-development datasets. The results confirm that the proposed framework can be applied to different road typologies within a real operational survey, supporting its use for road-asset inventory generation and maintenance-oriented mapping. However, this analysis should not be interpreted as a complete cross-regional generalization assessment. The survey was conducted within the same broad geographical context and does not fully cover differences in road-sign standards, pavement-marking conventions, environmental conditions, or infrastructure layouts that may occur in other regions or countries. For this reason, cross-regional validation on independent road networks remains an important future development. Further work will extend the testing campaign to additional geographical areas in order to assess the robustness and transferability of the framework under more diverse road, environmental, and regulatory conditions. To further assess the consistency of the final inventory, a manual count-based validation was performed on a 22 km subset of the independent survey, including 3 km of urban roads, 9 km of extra-urban roads, and 10 km of motorway sections. Before this comparison, frame-level detections were post-processed through a tracking and spatial consolidation procedure. Repeated detections of the same asset across consecutive frames were associated along the vehicle trajectory, and only the last useful position of each tracked asset within a predefined spatial area was retained as the final mapped inventory point. Therefore, the remaining discrepancies reported in Table 7 reflect inventory-level differences after tracking and duplicate merging.

Across the 22 km manually inspected sections, 279 real assets were counted, and 319 assets were included in the automatic inventory. Considering the absolute differences across road contexts and asset types, the cumulative absolute inventory error was 52 assets, corresponding to 2.36 assets/km and a relative inventory-count error of 18.64%. For vertical traffic signs, 216 real assets were counted, and 245 were predicted, with a cumulative absolute error of 31 assets, corresponding to 1.41 assets/km. For horizontal road markings, 63 real assets were counted, and 74 were predicted, with a cumulative absolute error of 21 assets, corresponding to 0.95 assets/km. The larger discrepancies observed for horizontal markings, especially in motorway sections, are mainly related to the visual and geometric characteristics of pavement assets in high-speed road environments. Elongated, repeated, dashed, transverse, or text-like markings may generate multiple detections or may be more difficult to consolidate into a single inventory object after post-processing. Therefore, the observed discrepancy is mainly associated with asset consolidation and residual duplicate-merging complexity, rather than with georeferencing alone.

## 6. Conclusions

This study presented a low-cost vision–GPS framework for the automated mapping of vertical traffic signs and horizontal road markings. The proposed methodology was designed to address two complementary categories of road infrastructure through a unified acquisition and processing workflow based on vehicle-mounted action cameras, GPS-based trajectory reconstruction, distance-aware frame extraction, deep learning recognition, and final georeferenced map generation. A key element of the framework is the adoption of a dual-pipeline architecture. Vertical traffic signs were processed as discrete roadside objects using object detection, while horizontal road markings were addressed through a segmentation-oriented approach, more suitable for elongated, irregular, and partially degraded pavement elements. This distinction allowed the specific geometric characteristics of each asset type to be handled more effectively within the same operational framework. The experimental results confirmed the effectiveness of the proposed approach. For vertical traffic signs, YOLO11m achieved the best overall performance among the evaluated models, with an overall F1-score of 0.92 and a mAP50 of 0.94 on the independent test set. For horizontal road markings, YOLO11m-seg provided the most accurate results, reaching an overall F1-score of 0.96 and a mAP50 of 0.98. These results demonstrate that the proposed low-cost acquisition setup, combined with dedicated dataset construction and model selection, can support reliable recognition of both vertical and horizontal road assets. Beyond detection performance, the main contribution of the study lies in the transformation of image-level outputs into georeferenced road-asset entities. By synchronizing visual detections with the GPS trajectory, the proposed workflow enables the automatic generation of spatial inventories that can support road infrastructure monitoring, maintenance planning, and digital road-network management. This aspect is particularly relevant for practical applications, since it connects visual recognition with operational mapping outputs that can be inspected and updated in a geographic environment. Some limitations remain. The detection of traffic lights and other visually variable vertical assets was less robust than that of standard traffic signs, mainly due to their smaller apparent size, heterogeneous appearance, and complex urban background conditions. For horizontal markings, the most challenging cases were associated with pedestrian crossings and triangular give-way markings, which may be affected by pavement texture, wear, partial occlusion, and reduced contrast. In addition, the georeferencing accuracy is influenced by the quality of the GPS trajectory and by the geometric assumptions used to associate detections with road positions. Future developments will focus on improving the robustness of the framework under more heterogeneous environmental and traffic conditions, extending the number of detectable asset classes, and refining the georeferencing procedure to enhance positional accuracy. Further work may also include temporal tracking of repeated detections, condition assessment of road assets, and larger-scale validation campaigns on different road networks. These developments would support the transition from automated asset detection toward operational digital inventories for intelligent transportation systems and road infrastructure management.

## Figures and Tables

**Table 1:** Quantitative comparison between distance-aware sampling and fixed-time sampling for the horizontal and vertical road-asset pipelines.

Pipeline	Strategy	N	Target (m)	Mean (m)	SD (m)	CV (%)	Short	Long	±20%
Horizontal markings	Distance-aware	7978	6.5	6.5	0.77	11.92	0	0	89.14
Horizontal markings	Fixed-time	7978	6.5	6.5	2.76	42.45	968	1020	35.39
Vertical signs	Distance-aware	2544	13	13.00	0.44	3.40	0	0	100.0
Vertical signs	Fixed-time	2544	13	13.01	4.76	36.57	216	194	57.09

N = number of sampled positions; SD = standard deviation of spacing; CV = coefficient of variation. For horizontal road markings, short intervals are <3.25 m and long intervals are >9.75 m. For vertical traffic signs, short intervals are <6.5 m and long intervals are >19.5 m. The ±20% column reports the percentage of intervals within ±20% of the target spacing.

**Table 2:** Distribution of annotated instances in the vertical traffic sign dataset across training, validation, and test subsets.

Class	Train	Val	Test	Total	Percentage
Danger	2003	571	286	2860	13%
Prohibition	2464	731	363	3558	16%
Priority	1033	298	147	1478	7%
Mandatory	1921	519	316	2756	12%
Stop	1233	361	166	1750	8%
Give-way	1459	411	185	2055	9%
No-stopping/No-parking	1370	399	251	2020	9%
No-entry	1206	361	187	1754	8%
Pedestrian crossing	2336	632	366	3334	15%
Traffic light	767	227	99	1093	5%

**Table 3:** Distribution of annotated instances in the horizontal road marking dataset across training, validation, and test subsets.

Class	Train	Val	Test	Total	Percentage
Give-way	627	116	143	886	11%
Left arrow	599	138	126	863	10%
Right arrow	613	120	142	875	10%
Stop	623	135	140	898	11%
Straight arrow	681	144	150	975	12%
Straight-left arrow	633	147	135	915	11%
Straight-right arrow	622	153	120	895	11%
Triangular give-way marking	671	135	145	951	11%
Pedestrian crossing	780	156	159	1095	13%

**Table 4:** Summary of the training configuration and evaluation setup adopted for the vertical and horizontal road-asset recognition pipelines.

Parameter	Vertical Sign Pipeline	Horizontal Marking Pipeline
Recognition task	Object detection	Instance segmentation and detection comparison
Input resolution	640 × 640 pixels	640 × 640 pixels
Annotation type	Bounding boxes	Polygonal masks
Evaluated models	YOLO11m, RT-DETR, Faster R-CNN	YOLO11m-seg, RT-DETR, Faster R-CNN
Dataset split	70% train, 20% validation, 10% test	70% train, 15% validation, 15% test
Main metrics	Precision, Recall, mAP50, mAP50-95, F1-score	Precision, Recall, mAP50, mAP50-95, F1-score
Final model selection	Test-set performance	Test-set performance

**Table 5:** Class-wise test performance of YOLO11m for vertical traffic sign detection.

Class	Instances	Precision	Recall	mAP50	mAP50-95	F1
Danger	286	0.96	0.92	0.97	0.89	0.94
Prohibition	363	0.96	0.96	0.98	0.92	0.96
Priority	147	1.00	0.95	0.97	0.96	0.97
Mandatory	316	0.98	0.84	0.95	0.86	0.90
Stop	143	0.99	1.00	0.99	0.99	0.99
Give-way	171	0.96	0.95	0.98	0.93	0.95
No-parking/No-stopping	230	0.98	0.74	0.92	0.85	0.84
No-entry	170	0.98	0.93	0.98	0.94	0.95
Pedestrian crossing	329	0.96	0.90	0.98	0.90	0.93
Traffic light	70	0.85	0.66	0.74	0.60	0.74
ALL	2225	0.96	0.88	0.94	0.88	0.92

**Table 6:** Class-wise test performance of YOLO11m-seg for horizontal road marking segmentation.

Class	Instances	Precision	Recall	Mask mAP50	Mask mAP50-95	F1
Give-way	142	0.972	0.973	0.992	0.94	0.97
Left arrow	126	0.981	0.976	0.989	0.967	0.98
Right arrow	142	0.993	0.983	0.995	0.978	0.99
Stop	140	0.98	0.964	0.983	0.92	0.97
Straight arrow	145	1	0.968	0.988	0.91	0.98
Straight-left arrow	135	0.991	1	0.995	0.959	0.99
Straight-right arrow	120	0.99	0.992	0.995	0.948	0.99
Triangular give-way marking	136	0.977	0.899	0.952	0.794	0.94
Pedestrian crossing	156	0.95	0.912	0.949	0.822	0.93
ALL	1242	0.98	0.96	0.98	0.91	0.96

**Table 7:** Manual inventory validation on a 22 km independent survey subset after tracking and duplicate merging. The table compares real and predicted assets and reports absolute, per-kilometre, and relative errors for vertical signs, horizontal markings, and the combined inventory.

Asset Type	Length (km)	Real Assets	Predicted Assets	Difference	Absolute Error	Error per km	Relative Error
Vertical signs	22	216	245	+29	31	1.41	14.35%
Horizontal markings	22	63	74	+11	21	0.95	33.33%
All assets	22	279	319	+40	52	2.36	18.64%

## Data Availability

The data presented in this study are available from the corresponding author upon reasonable request. Restrictions may apply due to the presence of georeferenced road-survey data and project-related constraints.

## Abbreviations

The following abbreviations are used in this manuscript:

CV	Coefficient of Variation
DFL	Distribution Focal Loss
EIS	Electronic Image Stabilization
FOV	Field of View
GNSS	Global Navigation Satellite System
GPS	Global Positioning System
HD	High Definition
IoU	Intersection over Union
ITS	Intelligent Transportation Systems
mAP	Mean Average Precision
RT-DETR	Real-Time Detection Transformer
SD	Standard Deviation of Spacing
YOLO	You Only Look Once
